# Composition and Metabolic Potential of Fe(III)-Reducing Enrichment Cultures of Methanotrophic ANME-2a Archaea and Associated Bacteria

**DOI:** 10.3390/microorganisms11030555

**Published:** 2023-02-22

**Authors:** Alexander I. Slobodkin, Nataliya M. Ratnikova, Galina B. Slobodkina, Alexandra A. Klyukina, Nikolay A. Chernyh, Alexander Y. Merkel

**Affiliations:** Winogradsky Institute of Microbiology, Research Center of Biotechnology, Russian Academy of Sciences, Leninskiy Prospect, 33, Bld. 2, 119071 Moscow, Russia

**Keywords:** methane, AOM, mud volcano, microbial communities, ANME, archaea, diversity, metagenome, MAG, *Desulfobulbaceae*

## Abstract

The key microbial group involved in anaerobic methane oxidation is anaerobic methanotrophic archaea (ANME). From a terrestrial mud volcano, we enriched a microbial community containing ANME-2a, using methane as an electron donor, Fe(III) oxide (ferrihydrite) as an electron acceptor, and anthraquinone-2,6-disulfonate as an electron shuttle. Ferrihydrite reduction led to the formation of a black, highly magnetic precipitate. A significant relative abundance of ANME-2a in batch cultures was observed over five subsequent transfers. Phylogenetic analysis revealed that, in addition to ANME-2a, two bacterial taxa belonging to uncultured *Desulfobulbaceae* and *Anaerolineaceae* were constantly present in all enrichments. Metagenome-assembled genomes (MAGs) of ANME-2a contained a complete set of genes for methanogenesis and numerous genes of multiheme c-type cytochromes (MHC), indicating the capability of methanotrophs to transfer electrons to metal oxides or to a bacterial partner. One of the ANME MAGs encoded respiratory arsenate reductase (Arr), suggesting the potential for a direct coupling of methane oxidation with As(V) reduction in the single microorganism. The same MAG also encoded uptake [NiFe] hydrogenase, which is uncommon for ANME-2. The MAG of uncultured *Desulfobulbaceae* contained genes of dissimilatory sulfate reduction, a Wood–Ljungdahl pathway for autotrophic CO_2_ fixation, hydrogenases, and 43 MHC. We hypothesize that uncultured *Desulfobulbaceae* is a bacterial partner of ANME-2a, which mediates extracellular electron transfer to Fe(III) oxide.

## 1. Introduction

Anaerobic methane oxidation (AOM) by microorganisms is a globally important process that prevents the release of a significant amount of methane into the atmosphere. It is estimated that more than 90% of the methane produced in the oceans is consumed by anaerobic oxidation [1]. The biological mechanisms of AOM are still insufficiently studied. One of the main problems is the difficulty of cultivating anaerobic methanotrophic archaea (ANME) and the microorganisms interacting with them. At present, not a single microorganism that performs AOM has been isolated in a pure culture. The habitats of ANME are diverse, and molecular methods show that they live in almost all ecosystems associated with methane emissions. Marine biotopes—cold methane seeps, sulfate-methane transition zones of sediments, water columns, and hydrotherms have been studied in the most detail [1,2]. In terrestrial ecosystems, ANME have been detected in mud volcanoes, freshwater sediments, and oilfield waters [1,3,4,5].

Different ANME groups can couple methane oxidation with the reduction of various electron acceptors, and the final stage of reduction can be carried out either by the ANME cells themselves or by a bacterial partner due to extracellular electron transfer. The coupling of AOM with the reduction of sulfate, nitrate, and nitrite, as well as Fe(III) and Mn(IV) compounds, has been reliably established. There are reports on the association of AOM with the reduction of humic acids, their artificial analogs (anthraquinone disulfonates, AQDS), chromate, selenate, and bromate [6,7,8,9,10,11].

The ability of microorganisms to couple AOM with the reduction of iron (Fe-AOM) has been determined in incubation experiments and confirmed by numerous geochemical data [12,13]. In marine sediments, ANME-2, capable of oxidizing methane using soluble forms of ferric iron, were identified [6]. Fe-AOM was demonstrated in bioreactors dominated by *Candidatus* Methanoperedens nitroreducens MPEBLZ [14]. Methane-dependent ferrihydrite reduction has been observed in artificial laboratory coculture systems consisting of AOM-denitrifying microorganisms and the iron-reducing bacterium *Shewanella oneidensis* MR-1 [15]. The produced ferrous iron was formed into minerals primarily composed of siderite with a small amount of vivianite and magnetite [15]. Ferrihydrite- and birnessite-dependent AOM could also be performed by other representatives of the “*Methanoperedenaceae*”—Ca. Methanoperedens ferrireducens, Ca. Methanoperedens manganicus and Ca. Methanoperedens manganireducens. Omics data suggest that in these archaea, multiheme cytochromes (MHC) extracellularly reduce Fe(III) and Mn(IV) during methane oxidation [16,17]. It has been shown that ANME-2a are the important microorganisms in iron-driven AOM in marine sediments [18].

The main microbiological studies of AOM have focused on the ANME; the diversity, distribution, and metabolism of the bacterial partners involved in this process are much less understood. None of the bacterial partners of ANME has been obtained in a pure culture, possibly because some of these organisms are obligate syntrophs. The syntrophic partners of the ANME belong to a number of phylogenetic groups of *Deltaproteobacteria*. The most common bacteria in natural and laboratory AOM sulfate-reducing consortia dominated by ANME-1 and ANME-2 are SEEP-SRB1, related to the *Desulfosarcina/Desulfococcus* group. SEEP-SRB2 bacteria belonging to *Desulfobacterales* are associated with ANME-2; bacteria related to *Desulfobulbus* are associated with ANME-3 [19,20,21]. Metagenomics has shown that the SEEP-SRB1 genomes contain MHC but do not have genes encoding periplasmic hydrogenases and formate dehydrogenases, typical for sulfate-reducing bacteria that live independently of ANME. *Candidatus* Desulfofervidus auxilii, a member of the clade “Hot Seep1” grows in association with thermophilic ANME-1 at 50–60 °C. This sulfate-reducing bacterium can be cultivated without ANME-1 using hydrogen as an electron donor [22]. Another thermophilic sulfate-reducing partner of ANME-1 at 70 °C is *Candidatus* Thermodesulfobacterium torris belonging to the class *Thermodesulfobacteria* [23]. Apart from “*Desulfobacterota”*, other bacterial groups that have been found to be significantly correlated with ANME are *Betaproteobacteria* and *Verrucomicrobia*, but the factors that determine interactions between these bacteria and archaea are not clear [24,25]. Currently, there is no information about bacteria associated with Fe-AOM.

Here, we report the cultivation of the stable enrichment cultures of Fe(III)/AQDS-reducing microorganisms with a significant relative abundance of ANME-2a. The phylogenetic composition of the enrichment cultures suggests that the probable partner of ANME-2a in Fe-AOM is an uncultured bacterium belonging to *Desulfobulbaceae*. Metagenomic analysis revealed the main metabolic features of ANME-2a and associated bacteria.

## 2. Materials and Methods

### 2.1. Sampling Sites Description, Sample Collection, and Chemical Analysis

**Figure 1:**
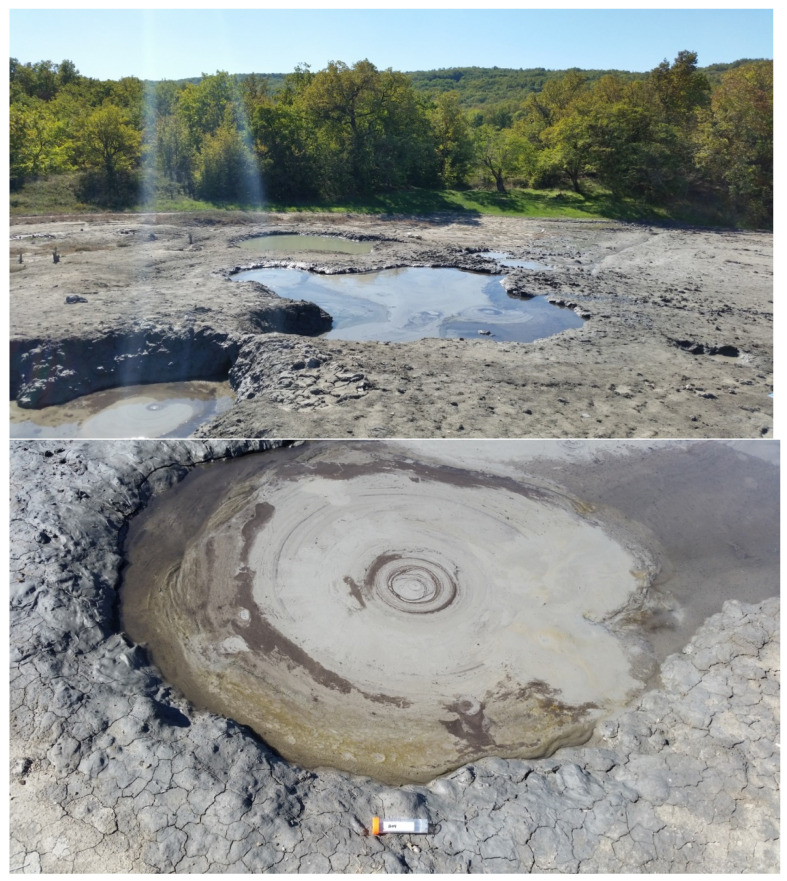
An image of the study site. A mud pool in the field of the terrestrial mud volcano Gladkovsky. The diameter of the pool is ca. 1.2 m.

A sample of mud was collected in September 2018, from a mud pool (about 1.2 m in diameter) located in the field of the terrestrial mud volcano Gladkovsky (45.005513 N, 37.723683 E), Krasnodar Krai, Russia. The sampling site was actively venting; large gas bubbles were breaking the mud at a high frequency (Figure 1). The sample was taken at a depth of 15–20 cm below the surface, placed into a sterile 50 mL tube, and transported to the laboratory, where it was prepared for microbiological and geochemical studies.

The temperature and pH of the sample were measured at the point of collection using a digital pH meter equipped with a thermocouple (AZ Instrument). Concentrations of chloride, sulfate, and nitrate were analyzed in water extracts by HPLC with a Stayer ion chromatograph (Aquilon) with an IonPack AS4-ASC column (Dionex) and conductivity detector; the eluent was bicarbonate (1.36 mM)/carbonate (1.44 mM), and the flow rate was 1.5 mLmin^−1^. The content of Fe(II) in the mud and enrichment cultures was determined after 24 h extraction in 0.6 M HCl, followed by a colorimetric assay of Fe(II) with 2,2-dipyridyl [26].

The mud temperature was 15 °C. The water had a pH of 7.43, a salinity of 30 g/L, and contained 390.6 mM of chloride, 0.59 mM of nitrate, and 0.1 mM of sulfate. Dissolved sulfide and nitrite were not detected (<0.01 mM). Previous studies showed that gas from the Gladkovsky terrestrial mud volcano is mainly composed of methane (94–96%) accompanied by dinitrogen and carbon dioxide [27].

### 2.2. Media and Cultivation

Enrichment was performed in an anaerobically prepared, bicarbonate-buffered, sterile liquid medium supplemented with ferrihydrite (poorly crystalline Fe(III) oxide; 90 mmol Fe(III)). Ferrihydrite was prepared as described in [28]. The medium contained (per liter distilled water): KH_2_PO_4_, NH_4_Cl, KCl, CaCl_2_•6H_2_O (0.33 g each), MgCl_2_•6H_2_O, 4.00 g; NaCl, 18.00 g; NaHCO_3_, 2.00 g, 1 mL trace element solution [29] and 1 mL vitamin solution [30]. The medium was prepared by boiling and cooling it under CO_2_ (100%) flow; afterward, NaHCO_3_ and vitamins were added. The medium was dispensed in 100 mL aliquots into 250 mL anaerobic bottles supplemented with rubber stoppers and screw caps; the head space was filled with CH_4_ (high purity grade, 100% *v/v*). No reducing agents were added to the medium. The medium was autoclaved at 1 atm, 121 °C for 1 h. The pH of the sterile medium was 7.5 at 25 °C. 9,10-anthraquinone-2,6-disulfonate (AQDS) from a sterile anoxic stock solution was added to a final concentration of 0.2 mM before inoculation.

**Figure 2:**
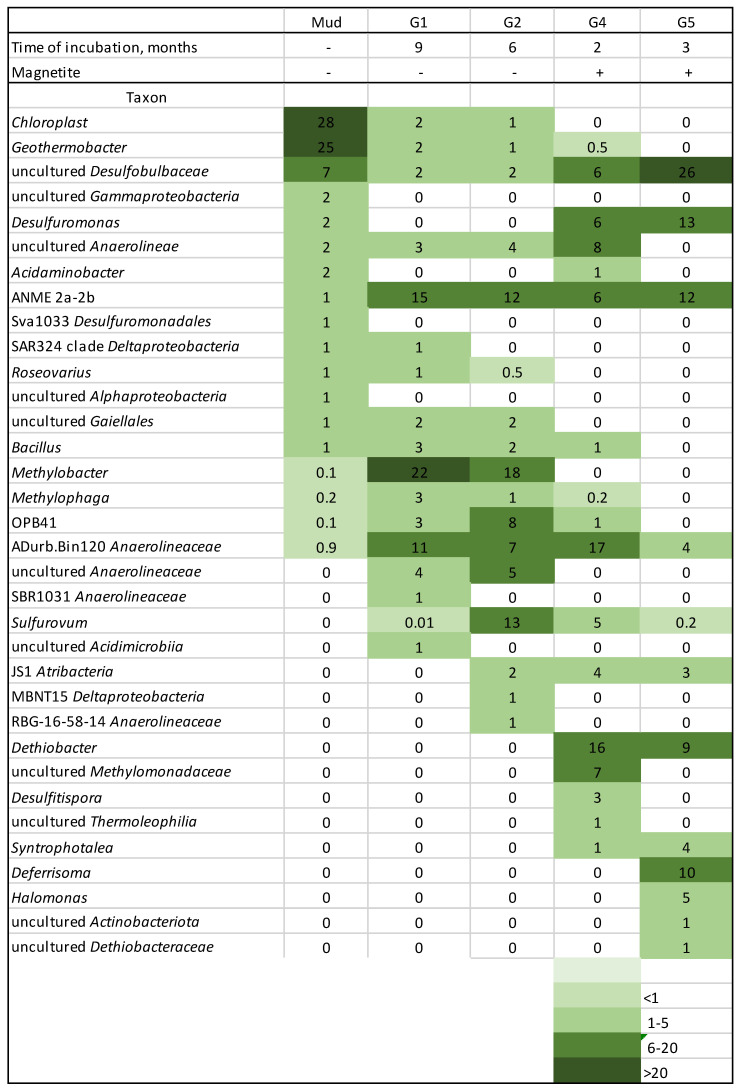
Taxa at the genus level, occurring at least once in a proportion of more than 1% in enrichment cultures. G3 was not sequenced.

An enrichment culture was initiated by inoculation of 50 mL of the mud into an anaerobic bottle containing 100 mL of sterile liquid medium supplemented with methane as a sole carbon and energy source, ferrihydrite as an electron acceptor, and AQDS as an electron-shuttling compound. The designation of this enrichment culture is G1. After 9 months of incubation, 30 mL of G1 (consisting of a mix of the liquid and solid phase) was transferred to 70 mL of the fresh medium with the same composition for further incubation (enrichment culture G2). The procedures of 30% transfers and incubations were repeated three more times at intervals of 2–6 months (enrichment cultures G3, G4, G5; Figure 2). All transfers and samplings of the cultures were performed with syringes and needles. Enrichments were incubated at 30 °C in the dark without agitation.

### 2.3. DNA Extraction, 16S rRNA Gene Amplicon and Metagenome Library Preparation, Sequencing, and Analysis

DNA from mud samples as well as from enrichment cultures was isolated using FastDNA Spin Kit for Soil according to the manufacturer’s protocol (MP Biomedicals, Santa Ana, CA, USA). The V4 region of the 16S rRNA amplicon libraries preparation, sequencing, and analysis were performed as previously described [31].

A shotgun metagenome library preparation and sequencing were done by BioSpark Ltd., Moscow, Russia using KAPA HyperPlus Library Preparation Kit (KAPA Biosystems, Wilmington, MA, USA) according to the manufacturer’s protocol and NovaSeq 6000 system (Illumina, San Diego, CA, USA) with the reagent kit, which can read 100 nucleotides from each end. Raw reads were processed with Cutadapt [32] and Trimmomatic [33] for adapter removal and quality filtering. Reads were processed in MetaWRAP [34] using MetaSPAdes [35] and MEGAHIT [36] for assembly, MaxBin 2 [37], MetaBAT 2 [38] and CONCOCT [39] for binning and Salmon [40] for coverage calculation. Bin completeness and contamination were evaluated using CheckM [41]. Taxonomies were assigned to each bin using GTDBtk [42]. MAGs were submitted for gene calling and annotations through the RAST [43]. Genes encoding metabolic functions were queried in RASTtk and IMG-MER using Blast search. Possible autotrophy has been investigated in all MAGs. We studied KEGG (Kyoto Encyclopedia for genes and genomes) biochemical maps for eight known carbon fixation pathways in each MAG [44,45,46,47]. The presence of S-layer domains in ANME MAGs was analyzed using InterProScan 5.60-92.0 [48]. All the sequencing data are deposited in NCBI BioProject PRJNA833668. MAGs Accession Numbers are JAMJFO000000000–jamjfv000000000.

## 3. Results and Discussion

### 3.1. Microbial Community of the Mud Volcano Gladkovsky

The microbial community in the sample from the mud volcano Gladkovsky, which was used as an inoculum for enrichment cultures, had a relatively low diversity with only 30 amplicon sequence variants (ASVs), a Shannon index of 2.2, and a Simpson index of 0.2. Chloroplast sequences were the most widely represented (Figure 2), which indicates an active process of photosynthesis on the mud surface. A quarter of the community was represented by the microorganisms within the *Geothermobacter* genus. Recently, a mesophilic microorganism of this genus has been described [49]. It has wide metabolic properties and is capable of anaerobic respiration using different electron acceptors: ferric citrate, Fe(OH)_3_, MnO_2_, AsO_4_^3−^, SO_4_^2−^, SeO_4_^2−^, S_2_O_3_^2−^, S^0^ and NO_3_^−^, and various organic compounds as well as H_2_ as primary electron donors. A significant proportion of the community (7%) was represented by the uncultured microorganism of the *Desulfobulbaceae* family. It was represented by only one ASV. A detailed analysis of the metabolic properties of this microorganism based on its genome analysis will be given below. Anaerobic methanotrophs were represented only by the ANME 2a–2b group and constituted only 1% of the community.

### 3.2. Cultivation and Taxonomic Composition of Enrichment Cultures

Our attempts to obtain stable enrichment cultures containing ANME from the Gladkovsky mud volcano using various electron acceptors (sulfate, nitrate, elemental sulfur) or without any acceptor did not yield positive results. After 9 months of incubation, only in enrichments amended with ferrihydrite, the relative abundance of ANME was higher than in the original sample. In enrichment cultures G4 and G5, ferrihydrite was converted to a black highly magnetic precipitate, probably magnetite, with Fe(II) content ca. 15 mM. In G1, G2, and G3, no formation of magnetic precipitate was observed, possibly due to the interaction of the formed Fe(II) with minerals still contained in the mud. To our knowledge, this is the first long-term cultivation of anaerobic microorganisms with methane oxidation resulting in a stable production of the magnetic precipitate.

In each enrichment culture, taxonomic diversity was determined at the end of incubation. Thirty-four taxa at the genus level occurred at least once in a proportion of more than 1% of the total prokaryotic abundance in all enrichment cultures (Figure 2). Only three genera with a relative abundance of more than 1% were constantly present in all enrichment cultures—uncultured *Desulfobulbaceae*, ANME 2a–2b, and ADurb.Bin120 (*Anaerolineaceae*). The relative abundance of ANME 2a–2b increased from 1% in the mud sample to 6–15% in the enrichment cultures. Controls incubated without methane showed a significantly lower abundance of ANME 2a–2b (0.7–0.9%). The relative abundance of uncultured *Desulfobulbaceae* and ADurb.Bin120 *Anaerolineaceae* in the enrichment culture G5 was 26% and 4%, respectively. Other bacteria present in G5 were represented by *Desulfuromonas* (13%), *Deferrisoma* (10%), *Dethiobacter* (9%), *Halomonas* (5%), and JS1 *Atribacteria* (3%).

Thus, we were able to cultivate a Fe(III)-reducing microbial community containing methanotrophic ANME 2a–2b archaea. Microbial reduction of ferrihydrite can occur either through direct contact between the cell and the mineral or through soluble AQDS as an electron-shuttling compound [50]. Four microorganisms in the G5 enrichment culture could potentially utilize ferrihydrite or AQDS as electron acceptors. Dissimilatory iron reduction was experimentally shown for representatives of the family *Desulfobulbaceae* and the genera *Desulfuromonas* and *Deferrisoma*, [51,52,53]. It is assumed that the ANME-2 also have the ability to reduce Fe(III) [5,6]. If the Fe-AOM in our enrichment cultures proceeds with the participation of a syntrophic bacterial partner, the most likely candidate for this role is the uncultured *Desulfobulbaceae,* which is constantly present in all enrichments. The family *Desulfobulbaceae* includes Fe(III) reducers as well as microorganisms capable of long-distance electron transport—e.g., cable bacteria *Candidatus* Electronema and *Candidatus* Electrothrix [54]. It has been reported that *Desulfobulbus* spp. form aggregates with ANME-2 and ANME-3 in marine mud volcanoes, and methane seep sediments where they are likely involved in sulfate-dependent AOM [24,55]. Alternatively, we can hypothesize that in our enrichment cultures, the uncultured *Desulfobulbaceae* is a consumer of exometabolites or microbial necromass, and it competes with ANME 2a–2b for Fe(III) or AQDS.

### 3.3. MAGs General Characteristics and Phylogenetic Identification

Metagenomic sequencing of enrichment culture G2 was performed. Eight MAGs with >75% completeness and <1.5% contamination were recovered from the metagenome. The MAGs statistics, including the number of contigs, genome size, the presence of 16S rRNA gene, and relative abundance calculated based on coverage estimation, are shown in Table 1.

**Figure 3:**
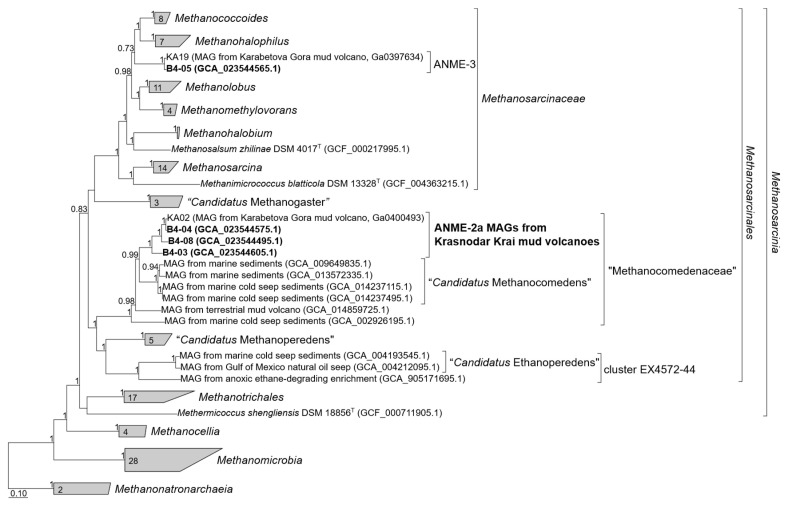
Phylogenomic placement of ANME MAGs based on concatenated partial amino acid sequences of 53 archaeal single copy conserved marker genes with taxonomic designations according to the GTDB. The tree was built using the IQ-TREE 2 program [58] with fast model selection via ModelFinder [59] and ultrafast bootstrap approximation [60], as well as an approximate likelihood-ratio test for branches [61]. Bootstrap consensus tree is shown with values above 70% placed at the nodes. Bar, 0.1 changes per position.

Enrichment culture G2 contained two large populations of ANME-2a represented by MAG B4-03 and MAG B4-04 with a relative abundance in the metagenome of 53.8% and 30.2%, respectively. According to phylogenomic reconstruction based on 53 archaeal single copy conserved marker genes [56], all ANME-2a genomes from Krasnodar Krai mud volcanoes form a separate genus-level lineage mostly related to recently named genus “*Candidatus* Methanocomedens” [57] (Figure 3). The difference between the «Krasnodar group» and Methanocomedens is also confirmed by average nucleotide identity (ANI) analysis: the average ANI within the Methanocomedens group is 93.4%, the average ANI within the «Krasnodar group» group is 87.6%, while the average ANI between the genomes of these groups is 83.3%. The same picture is for AAI: 90.4%, 80.4%, and 72.3%, respectively. The MAG of ANME-3 microorganism is closely related to MAG from the Karabetova Gora mud volcano, which was described in detail in our previous work [5].

Two MAGs were not identified by GTDB-Tk up to the genus level: MAG B4-07 was identified as a member of the *Anaerolineae,* and MAG B4-01—as a member of the *Desulfobulbaceae*. A more precise phylogenetic analysis is shown in Supplementary Figure S1A,B. MAG B4-07 is a part of the order-level lineage JAAYZQ01 from *Anaerolineae* class surrounded by MAGs from various ecotopes; MAG B4-01 is mostly related to UBA10518 and BMS3Abin13 uncultured clusters of the genus-level within *Desulfobulbaceae* and surrounded by MAGs from subsurface ecotopes and ecotopes associated with the geothermal activity.

For the analysis of the metabolic potential encoded in the genomes, we choose two MAGs (B4-03 and B4-04) belonging to the ANME-2a, which dominates enrichment, and one MAG (B4-01) belonging to the uncultured *Desulfobulbaceae*, a probable bacterial partner of ANME. These three MAGs represent ca. 90% of metagenome abundance and have completeness > 85%.

### 3.4. Insights into Carbon and Energy Metabolism of ANME-2a

MAGs of ANME-2a, B4-03, and B4-04 contained genes encoding the central steps of the methanogenic pathway. Genes of formyl-methanofuran dehydrogenase (*fmdABCDEFG*), formyl-methanofuran tetrahydromethanopterin formyl transferase (*ftr*), methenyl-tetrahydromethanopterin cyclohydrolase (*mch*), methylene-tetrahydromethanopterin dehydrogenase (*mtd*), methylene- tetrahydromethanopterin reductase (*mer*), tetrahydromethanopterin methyltransferase (*mtrABCDEFGH*), and methyl-coenzyme M reductase (*mcrABCD*) were identified. MAG B4-04 lacked the *mcrA* gene, while *mcrBCD* genes were present. Likely, the *mcrA* gene was missed during genome assembly since the *mcr* gene cluster is located at the very end of the contig.

Genes encoding soluble F_420_-dependent (Frh) and F_420_-nonreducing (Mvh) hydrogenases, as well as membrane-bound ferredoxin-dependent (Ech) and methanophenazine-dependent (Vho) hydrogenases, common in hydrogenotrophic and methylotrophic methanogens, were absent in MAG B4-03 and MAG B4-04. Surprisingly, MAG B4-04 encoded protein, which was annotated by RAST as a large subunit of uptake hydrogenase and by PGAP as a large subunit of nickel-dependent hydrogenase (MCL7474638). According to the HydDB classifier [62], this protein belongs to Group 2: Alternative and sensory H_2_-uptake [NiFe] hydrogenases. The absence of N-terminal signal peptides and transmembrane helices suggests that it is located in the cytoplasm. The gene of large hydrogenase subunit is a part of the cluster, which codes for uptake hydrogenase small subunit precursor (MCL7474639), four hydrogenase assembly proteins HypDEFC (MCL7474633-36) and hydrogenase maturation protease (MCL7474637). Genes for [FeFe] and [NiFe] hydrogenases were identified in ANME-1 and *Methanoperedenaceae* [23,57,63,64], but as far as we know, the presence of hydrogenases in the genomes of ANME-2 has not been reported previously. Our finding suggests that representatives of ANME-2 are potentially able to perform hydrogenotrophic methanogenesis.

The enzymes responsible for the utilization of methanol, methylamines, and methoxylated aromatic compounds—methanol-corrinoid methyltransferases, mono-, di- and three-methylamine methyltransferases, corrinoid proteins, and methanol dehydrogenase were not encoded in either ANME genomes (Supplementary Table S1).

Electrons generated from methane oxidation could be transferred to the lipid-soluble electron acceptors, methanophenazine, or menaquinone, via the energy-conserving Fpo, Hdr, and Rnf complexes, which oxidize F_420_H_2_, CoM-SH + CoB-SH, and reduced ferredoxin, respectively. Genes of F_420_H_2_:phenazine oxidoreductase (Fpo subunits ABCD), cytoplasmic (HdrABC), and membrane bound (HdrDE) coenzyme B-coenzyme M heterodisulfide reductase were present in both MAGs. Genes of Na^+^-translocating, ferredoxin:NAD oxidoreductase (Rnf subunits ABCDGE) were identified only in MAG B4-03.

The nitrate reductase complex responsible for dissimilatory nitrate reduction in *Methanoperedens*-like archaea [65] was not encoded in either the B4-03 or B4-04 genomes. No homologs of *nar, nap, nrf, nir, nor,* or *nos* genes were found. Genes involved in dissimilatory sulfate reduction (dsrAB, dsrC, qmoA,B,C, aprAB, sat) were also absent. Hence, most likely, both archaea do not use nitrate or sulfate as electron acceptors.

The presence of multiheme c-type cytochromes (MHCs) has been reported for ANME-1, ANME-2, and ANME-3 [5,57,66]. Among the members of Ca. Methanoperedenaceae, MHC are involved in the reduction of insoluble Fe(III) or Mn(IV) oxides [16,17]. We identified 8 MHCs (with a number of hemes > 4) in MAG B4-03 and 20 MHCs in MAG B4-04 (Table 2, Supplementary Table S2). Seven MHCs in the B4-03 genome and 17 MHCs in the B4-04 genome were predicted to contain C-terminal transmembrane helices (TMHs), indicating they were localized on the external side of the cell membrane. The number of CxxCH heme c binding motifs in a single MHC varied from 5 to 32 in B4-03 and from 4 to 45 in B4-04. Genes encoding exceptionally large MHCs with 20 and 80 heme-binding sites were noted previously in several ANME genomes. The presence of an S-layer domain in some of these cytochromes suggests their use for electron transfer through this outermost layer [57]. In B4-03 and B4-04 MAGs two MHCs contained S-layer family duplication domain IPR006457: MCL7415845 (32 hemes, length 1647 aa) and MCL7475970 (33 hemes, length 1704 aa), indicating a role of these proteins as putative S-layer conduits.

B4-03 and B4-04 genomes contained genes encoding Acd, - the alpha and beta chains of acetyl-CoA synthetase (ADP-forming) (MCL7414629 and MCL7474717), an archaeal enzyme that catalyzes the conversion of acetyl-CoA to acetate coupled with the conversion of ADP to ATP. Thus, B4-03 and B4-04 have the possibility to produce acetate from acetyl-CoA formed by CO dehydrogenase/acetyl-CoA synthase, which is encoded in both MAGs (MCL7414833 and MCL7475685). This acetate could be excreted from the cell and utilized by other microorganisms as it was suggested for ANME-2 and sulfate-reducing bacteria [67].

**Figure 4:**
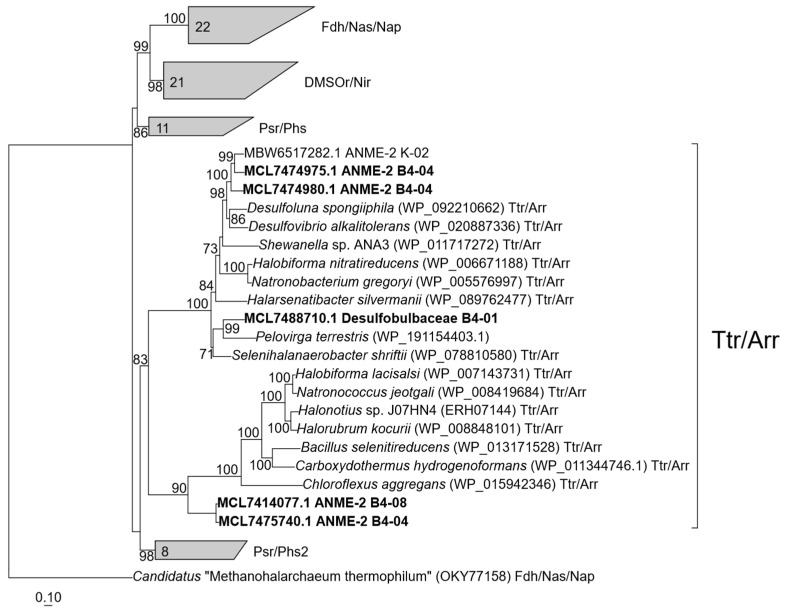
Phylogenetic tree of CISM catalytic subunits A. Totally 84 sequences were taken for the analysis. Abbreviations: DMSO/Nar, DMSO/nitrate reductase family; Fdh/Nas/Nap/, assimilatory nitrate reductase/formate dehydrogenase family; Psr/Phs, polysulfide/thiosulfate reductase family; Ttr/Arr, tetrathionate/arsenate reductase family. Sequences studied in this article are shown in bold. The tree was built using the IQ-TREE 2 program [58] with fast model selection via ModelFinder [59] and ultrafast bootstrap approximation [60], as well as an approximate likelihood-ratio test for branches [61]. Bootstrap consensus tree is shown with values above 70% placed at the nodes. Bar, 0.1 changes per position.

Unexpectedly, MAG B4-04 encoded proteins, which were annotated by RAST as two subunits of respiratory arsenate reductase ArrAB. PGAP annotated these proteins as molybdopterin-dependent oxidoreductase (MCL7474975 and MCL7474980—two copies of putative ArrA) and 4Fe-4S dicluster domain-containing protein (MCL7474976, putative ArrB). In the amino acid sequences of these proteins, we found the CX2CX3C motif, which is characteristic of ArrA and presumably anchors the [4Fe-4S] cluster, and the CX2CX2CX3C motif, which usually binds the [Fe-S] centers in ArrB. The putative ArrA had a high amino acid sequence identity with the corresponding subunit of the well-studied respiratory arsenate reductases from *Chrysiogenes arsenatis* [68] and *Shewanella* strain ANA-3 [69] (53% and 58%, respectively). Phylogenetic analysis showed that ArrA from MAG B4-04 falls in the Trr/Arr cluster (tetrathionate/arsenate reductase family) in the CISM superfamily (complex iron-sulfur molybdoenzymes) (Figure 4). To our knowledge, the presence of Arr genes in ANME was reported only for one MAG belonging to *Candidatus* Methanoperedens nitroreducens [64]. Our finding suggests that ANME-2 may also use Ar(V) as a terminal electron acceptor during the anaerobic oxidation of methane or another electron donor.

In summary, the genome of B4-04 is 20% bigger than the genome of B4-03; it encodes H_2_-uptake [NiFe] hydrogenase, respiratory arsenate reductase, and twice more multiheme c-type cytochromes. Opposite, the genome of B4-03 contains the Rnf electron transport complex, which is absent in B4-04.

### 3.5. Insights into Carbon and Energy Metabolism of Uncultured Desulfobulbaceae

MAG B4-01 contained all the genes necessary for autotrophic CO_2_ fixation through the reductive acetyl-CoA (Wood–Ljungdahl) pathway. The genes encoding five subunits of the CO dehydrogenase/acetyl-CoA synthase complex and bifunctional 5,10-methylenetetrahydrofolate dehydrogenase/5,10-methenyltetrahydrofolate cyclohydrolase were organized in one cluster. Formate dehydrogenase (EC 1.2.2.1) and formyltetrahydrofolate synthetase (EC 6.3.4.3) participating in the methyl branch of the Wood–Ljungdahl pathway were placed separately. GenBank accession numbers for the detected annotated putative proteins are given in Supplementary Table S3. The key enzymes of other known bacterial carbon fixation pathways, namely, ribulose-1,5-bisphosphate carboxylase/oxygenase (Calvin–Benson cycle), ATP-citrate lyase and citryl-CoA lyase (two variants of the reductive tricarboxylic acid cycle), and glycine reductase complex (the reductive glycine pathway) were absent.

The genome contained a complete set of genes of glycolysis/gluconeogenesis via Embden–Meyerhof–Parnas pathway, including the gene of 6-phosphofructokinase, which catalyzes the irreversible reaction of glycolysis. The gene of the second irreversible reaction of glycolysis, pyruvate kinase (EC 2.7.1.40), was not found. Instead, the genome harbored numerous genes of phosphoenolpyruvate synthase (EC 2.7.9.2.), pyruvate phosphate dikinase (EC 2.7.9.1.), and bifunctional phosphoenolpyruvate synthase/pyruvate phosphate dikinase that enables the reversible transformation of phosphoenolpyruvate to pyruvate. Thus, this pathway can operate in both directions. Complete sets of genes for the pentose phosphate pathway and citric acid cycle (TCA) were found in the genome. These data suggest that the studied microorganism is capable of autotrophic as well as heterotrophic metabolism.

MAG B4-01 contained three different [NiFe] hydrogenases. One of them mostly resembled respiratory H_2_-uptake [NiFe] hydrogenase belonging to Group 1b. Hydrogenases of Group 1b participate in hydrogenotrophic respiration using sulfate, fumarate, nitrate, metals, and azo compounds as terminal electron acceptors and are widespread among obligately and facultatively anaerobic *Desulfobacterota* and *Campilobacterota* [70]. Genes *hynABC* encoded large, small, and cytochrome *b* membrane subunits of hydrogenase. Genes *hypC, hypD, hypE,* and *hypF,* responsible for the production and delivery of the iron center along with its diatomic ligands, and genes *hypA* and *hypB,* required for nickel insertion, were present in the same cluster. MAG B4-01 also encoded four subunits of sulfhydrogenase complexes (*hydADGB*) where the alpha and delta subunits function as hydrogenases and the beta and gamma subunits function as sulfur reductase. In addition, MAG B4-01 harbored a six-gene cluster resembling *hyc* or *hyf* operon encoding hydrogenase 3 or 4 from *Escherichia coli* K-12. In *E. coli,* both hydrogenases function with formate dehydrogenase H (FDH-H) as an energy-conserving formate hydrogenlyase that couples formate oxidation to H_2_ evolution. Homologs of large and small catalytic subunits of hydrogenase 3 or 4 in MAG B4-01 had 26% and about 40% of amino acid sequence identity with respected subunits (products of *hycE*/*hyfG* and *hycG*/*hyfI*) of hydrogenase 3 and 4 from *E. coli* K-12.

An energy-conserving electron-transport chain in MAG B4-01 was represented by Na^+^-translocating NADH-quinone reductase Na^+^-NQR (EC 7.2.1.1), H^+^ F_1_F_o_-ATP synthase (EC 7.1.2.2), and Na^+^/H^+^ antiporters. Genes of six subunits (*nqrABCDEF*) of Na^+^-NQR, two clusters of genes encoding for subunits of H^+^ F_1_F_o_-ATP synthase, and two clusters with genes for Na^+^/H^+^ antiporter subunits EGBBCDAH and ADCBG were found. Na^+^-NQR catalyzes the transfer of electrons from NADH to ubiquinone and uses the free energy released by the redox reaction to pump sodium. The sodium motive force generated by the Na^+^-NQR may be used for the establishment of a proton gradient via Na^+^/H^+^ antiporters that then drives the synthesis of ATP via an H^+^ F_1_F_o_-ATP synthase. Additionally, genes encoding for subunits IKEABD of V-type ATP synthase were identified.

MAG B4-01 contained the complete set of genes involved in dissimilatory sulfate reduction, including sulfate adenylyltransferase, *sat*, and adenylyl-sulfate reductase *aprAB,* manganese-dependent inorganic pyrophosphatase *ppaC*, dissimilatory sulfite reductase *dsrABD*, and *dsrC,* which was located separately. Genes coding for the electron transfer complexes, *dsrMKJOP* and *qmoABC,* were also present. No known genes involved in sulfur compound oxidation, in particular, *soeABC* or *soxXYZABCD,* were found.

MAG B4-01 encoded two arsenate-reducing enzymatic systems: Arr—for As(V) respiration and Ars—for arsenic resistance. Putative respiratory arsenate reductase subunits A and B had 51% and 45% of amino acid sequence identity with respective subunits of Arr from *Shewanella* strain ANA-3 [69]. High protein sequence identity was also observed with putative ArrAB encoded in MAG B4-04 of ANME-2a, 57% and 47%, respectively (see Section 3.4 and Figure 4). A detoxification system for As(V) reduction and extrusion (Ars) was indicated in MAG B4-01 by a cytosolic arsenate reductase ArsC (EC 1.20.4.4) and arsenite permease of the Acr3 family that catalyzes the efflux of arsenite from the cells.

MAG B4-01 contained a gene cluster encoding proteins involved in nitrogen fixation. These include alfa and beta chains of nitrogenase (molybdenum-iron type) (EC 1.18.6.1), proteins related to nitrogenase cofactors, and nitrogen regulatory proteins P-II. *Nif ENB* operon, which is responsible for the formation of a functional Mo-Fe-co catalytic site for nitrogenase, was located separately. Genes for ammonium transporters were also present. Genes for known enzymes of dissimilatory nitrate reduction were missing, with the exception of *norBC* and *norQD* encoding for nitric-oxide reductase and nitric-oxide reductase activation protein, respectively.

In MAG B4-01, we identified 43 MHCs (having >4 hemes) (Table 2, Supplementary Table S4). Thirty-nine MHCs were predicted to contain C-terminal transmembrane helices (TMHs), indicating their localization outside of the cytoplasm. The number of CxxCH heme c binding motifs in a single MHC varied from 4 to 39. Large numbers of MHCs are typical for microorganisms that are capable of extracellular electron transfer; for example, the genomes of well-studied Fe(III)-reducing bacteria *Geobacter sulfurreducens* and *Shewanella oneidensis* encode 75 and 39 MHCs, respectively [71].

Based on genome analysis, we can expect that the bacterium thriving in our enrichment culture belonging to uncultured *Desulfobulbaceae* is capable of lithoautotrophic growth with molecular hydrogen, dinitrogen fixation and dissimilatory sulfate, arsenate, and Fe(III) reduction. It also might be able to utilize organic compounds, e.g., glucose, pyruvate, and intermediates of the TCA cycle by anaerobic respiration or fermentation.

## 4. Conclusions

We succeeded in long-term batch cultivation of the enrichment cultures transforming ferrihydrite into magnetic precipitate in the presence of AQDS under a CH_4_ atmosphere. The enrichments contained two different species of anaerobic methanotrophic archaea belonging to the ANME-2 group and uncultured representatives of *Desulfobulbaceae* and *Anaerolineaceae*. We have identified the respiratory arsenate reductase (Arr) genes in the ANME genomes, which indicates the possibility of ANME-2 using Ar(V) as a terminal electron acceptor during the anaerobic oxidation of methane or another electron donor. One of the ANME-2 MAGs encodes H_2_-uptake [NiFe] hydrogenase. The presence of hydrogenases of this type in the genomes of ANME-2 has not been reported previously and suggests that members of this archaeal group are able to perform hydrogenotrophic methanogenesis. The metabolic machinery of two ANME-2a species coexisting in the enrichments differs in the presence or absence of the Rnf electron transport complex and in the number of multiheme c-type cytochromes. It is likely that these two methanotrophic archaea employ different mechanisms of AOM with regard to the terminal electron acceptor and the involvement of the syntrophic partner. The mechanism of Fe(III) reduction in our enrichments is not clear and may include the reduction of ferrihydrite or AQDS by both ANME-2 cells and the cells of the associated bacteria. The most probable candidate to be the iron-reducing syntrophic partner is the uncultured *Desulfobulbaceae* bacterium. Its genome contains 43 genes encoding MHC. Our data show that the association between ANME-2 and *Desulfobulbaceae* is relevant not only for sulfate-dependent AOM but also for Fe-AOM. Overall, our results demonstrate the involvement of ANME-2 in microbial processes that occur during dissimilatory Fe(III) reduction and extend the knowledge about the possible functions and interactions of ANME-2 in AOM microbial communities.

## Figures and Tables

**Table 1:** Overview of all genome bins >50% complete and with <5% contamination.

Bin ID	Domain	Taxon	Abundance %	Completeness %	Contamination %	# Contigs	Genome Size, Mbp	16S rRNA Gene, bp
B4-03	A	ANME-2a (HR1)	53.83	97.05	0.33	153	1.63	-
B4-04	A	ANME-2a (HR1)	30.20	94.11	0.65	144	1.98	-
B4-05	A	ANME-3	6.13	84.72	0.65	82	1.77	1426
B4-02	B	*Methylobacter marinus*	1.98	98.76	0.69	138	4.69	-
B4-08	A	ANME-2a (HR1)	1.97	98.40	0.98	439	2.34	-
B4-07	B	*Anaerolineae*	1.39	92.48	1.36	541	4.53	-
B4-01	B	*Desulfobulbaceae*	1.34	85.54	1.16	161	3.85	914
B4-06	A	*Halalkalicoccus*	0.60	78.93	1.02	910	2.54	-

**Table 2:** Multiheme c-type cytochromes (MHCs), encoded in MAGs of ANME and uncultured *Desulfobulbaceae*.

	B4-03 (ANME-2a)	B4-04 (ANME-2a)	B4-01 Uncultured *Desulfobulbaceae*
Number of MHCs	8	20	43
Number of MHCs with TMHs	7	17	27
Number of MHCs with CxxCH >10	4	11	18
Maximal number of CxxCH in single MCH	32	45	39
Length, aa (max–min)	211–1647	198–2234	153–2354

## Data Availability

Not applicable.

## Supplementary Materials

The following supporting information can be downloaded at: https://www.mdpi.com/article/10.3390/microorganisms11030555/s1, Table S1: Methyl genes; Table S2: Cytochromes ANME; Table S3 Gene locus; Table S4: Cytochromes B4-01. Figure S1: Phylogenetic analysis of the MAGs B4-01 and B4-07.
