# Supplementary material for: Composition and Metabolic Potential of Fe(III)-Reducing Enrichment Cultures of Methanotrophic ANME-2a Archaea and Associated Bacteria
Source: Microorganisms. 2023 Feb 22;11(3):555. doi: 10.3390/microorganisms11030555 (PMC10052568; doi:10.3390/microorganisms11030555)
Supplement: Supplementary file 1 [file microorganisms-11-00555-s001.zip › Supplementary Figure S1.pdf]

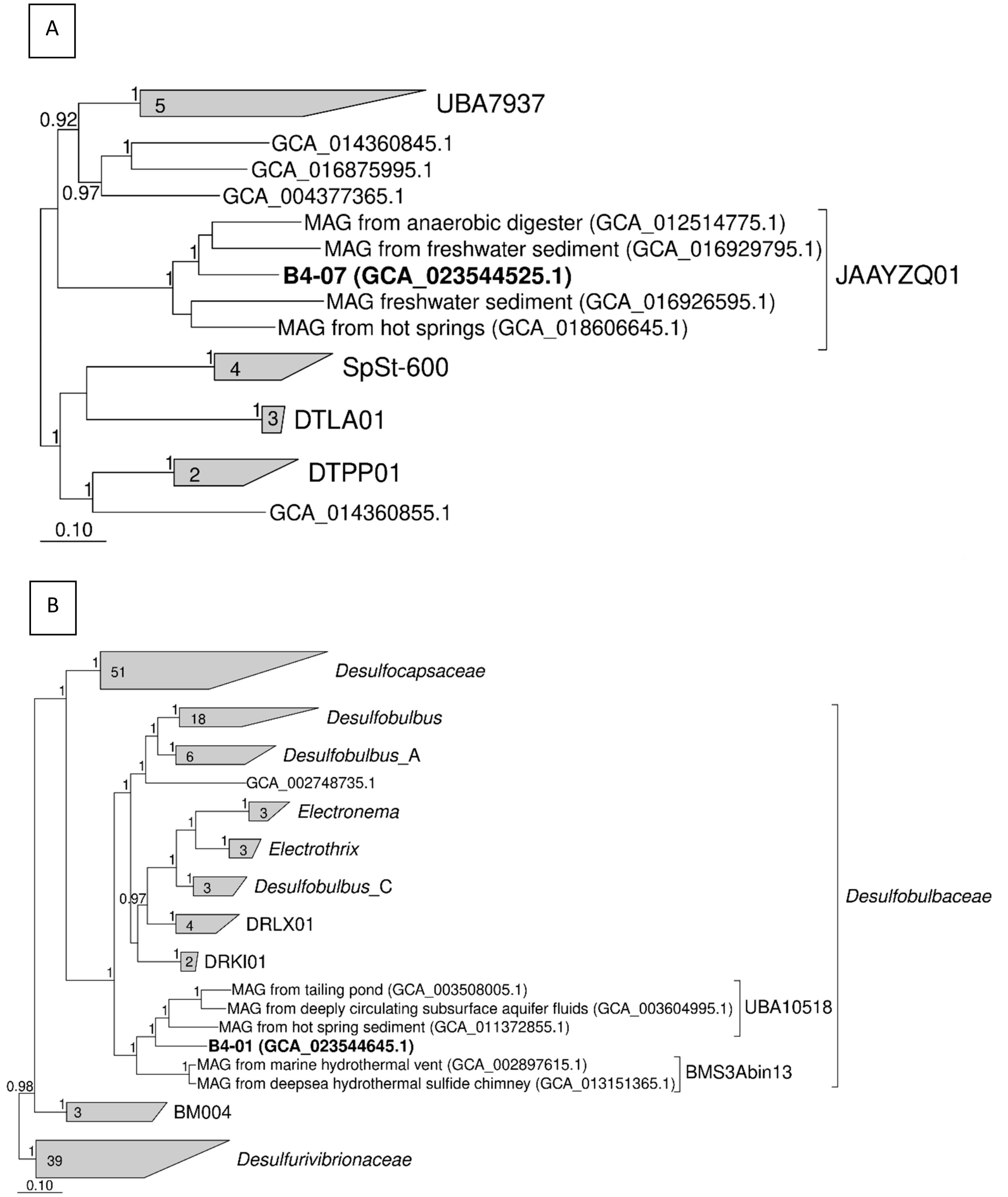

Supplementary Figure S1. Phylogenomic placement of B4-07 (A) and B4-01 (B) MAGs based on concatenated partial amino acid sequences of 120 bacterial single copy conserved marker genes with taxonomic designations according to the GTDB. The trees were built using the GTDB-Tk v2.1.1 (de_novo_wf) (Chaumeil et al., 2022). Bootstrap consensus tree is shown with values above 90% placed at the nodes. Bar, 0.1 changes per position.

1. Chaumeil, P.A.; Mussig, A.J.; Hugenholtz, P.; Parks, D.H.; GTDB-Tk v2: memory friendly classification with the genome taxonomy database. *Bioinformatics* **2022**, 38, 5315-5316. doi: 10.1093/bioinformatics/btac672.
