# Supplementary material for: Composition and Metabolic Potential of Fe(III)-Reducing Enrichment Cultures of Methanotrophic ANME-2a Archaea and Associated Bacteria
Source: Microorganisms. 2023 Feb 22;11(3):555. doi: 10.3390/microorganisms11030555 (PMC10052568; doi:10.3390/microorganisms11030555)
Supplement: Supplementary file 1 [file microorganisms-11-00555-s001.zip › Supplementary Table S2 Cytochromes ANME.pdf]

Supplementary Table S2. Multiheme c-type cytochromes encoded in MAGs B4-03 and B4-04 (ANME-2a)

B4-03					B4-04				
Locus tag	RAST	Length, aa	Number of heme *c*-binding motifs	Predicted cellular location	Locus tag	RAST	Length, aa	Number of heme *c*-binding motifs	Predicted cellular location
MCL7415845	peg.1740	1647	32	TM	MCL7474958	peg.2258	454	5	TM
MCL7414464	peg.875	368	16	TM	MCL7476062	peg.1456	423	8	TM
MCL7414463	peg.874	211	9	TM	MCL7476351	peg.1913	512	7	TM
MCL7414598	peg.176	373	16	TM	MCL7475379	peg.448	447	9	TM
MCL7415930	peg.2	334	6	TM	MCL7475737	peg.1028	304	11	periplasm
MCL7415476	peg.1339	481	6	TM	MCL7474552	peg.968	367	15	TM
MCL7416150	peg.651	1241	5	cytoplasm	MCL7475773	peg.1067	295	8	periplasm
MCL7414804	peg.467	313	10	TM	MCL7474596	peg.1273	1013	15	TM
					MCL7475154	peg.201	286	13	TM
					MCL7475111	peg.158	373	16	TM
					MCL7475189	peg.239	1860	13	TM
					MCL7475280	peg.338	836	17	TM
					MCL7475211	peg.262	2234	45	TM
					MCL7474551	peg.967	213	8	TM
					MCL7475970	peg.1271	1704	33	TM
					MCL7475771	peg.1065	198	4	TM
					MCL7476502	peg.2253	277	7	cytoplasm
					MCL7474817	peg.1999	310	10	TM
					MCL7476338	peg.1899	333	6	TM
					MCL7474900	peg.2144	1226	15	TM

*TM – transmembrane
