# Supplementary material for: Composition and Metabolic Potential of Fe(III)-Reducing Enrichment Cultures of Methanotrophic ANME-2a Archaea and Associated Bacteria
Source: Microorganisms. 2023 Feb 22;11(3):555. doi: 10.3390/microorganisms11030555 (PMC10052568; doi:10.3390/microorganisms11030555)
Supplement: Supplementary file 1 [file microorganisms-11-00555-s001.zip › Supplementary Table S4 Cytochromes B4-01.pdf]

Supplementary Table S4. Multiheme c-type cytochromes encoded in MAG B4-01

Locus tag	RAST	Length, aa	Number of heme *c*-binding motifs	Predicted cellular location
MCL7489785	peg.327	661	26	TM
MCL7487723	peg.544	486	5	periplasm
MCL7487805	peg.646	591	11	TM
MCL7487986	peg.855	388	6	TM
MCL7486557	peg.911	485	5	TM
MCL7486741	peg.1549	331	12	periplasm
MCL7489107	peg.2749	512	9	periplasm
MCL7489124	peg.2769	491	5	TM
MCL7489194	peg.2944	1621	32	TM
MCL7487168	peg.3064	879	8	TM
MCL7489334	peg.3203	214	5	TM
MCL7487347	peg.3561	339	9	TM
MCL7489301	peg.3163	307	10	TM
MCL7489603	peg.3685	576	14	TM
MCL7489784	peg.326	197	7	TM
MCL7487979	peg.845	405	16	cytoplasm
MCL7488685	peg.2020	349	8	periplasm
MCL7489246	peg.3006	1008	32	periplasm
MCL7488076	peg.1144	305	12	periplasm
MCL7488953	peg.2575	205	4	TM
MCL7489214	peg.2968	2354	39	cytoplasm
MCL7488832	peg.2332	259	7	TM
MCL7489042	peg.2679	184	4	TM
MCL7488217	peg.1300	2196	13	TM
MCL7489249	peg.3009	2072	35	TM
MCL7488077	peg.1145	267	12	TM
MCL7489606	peg.3689	265	12	periplasm
MCL7489274	peg.3040	275	11	TM
MCL7487026	peg.2483	176	4	TM
MCL7489248	peg.3008	709	4	TM
MCL7486914	peg.2110	637	10	cytoplasm
MCL7487971	peg.834	177	5	periplasm
MCL7487543	peg.255	474	8	periplasm
MCL7487438	peg.3756	227	6	TM
MCL7487720	peg.540	487	9	periplasm
MCL7488952	peg.2574	214	4	TM
MCL7489062	peg.2700	155	4	TM
MCL7487248	peg.3314	153	4	TM
MCL7487466	peg.3790	1294	13	periplasm
MCL7488558	peg.1875	481	4	cytoplasm
MCL7487998	peg.873	166	4	TM
MCL7487433	peg.3745	492	8	periplasm
MCL7488635	peg.1960	209	5	TM

*TM – transmembrane
